# Sulfur analyses and mineralogical data in the preliminary mine waste characterization

**DOI:** 10.1007/s10661-022-10094-9

**Published:** 2022-05-18

**Authors:** Teemu Karlsson, Päivi M. Kauppila, Marja Lehtonen, Lena Alakangas, Tommi Kauppila

**Affiliations:** 1 Circular Economy Solutions, Geological Survey of Finland, Neulaniementie 5, 70211 Kuopio, Finland; 2 Department of Civil, Environmental and Natural Resources Engineering, Division of Geosciences and Environmental Engineering, Luleå University of Technology, 97187 Luleå, Sweden; 3 Circular Economy Solutions, Geological Survey of Finland, Vuorimiehentie 2, 02151 Espoo, Finland

**Keywords:** Acid mine drainage, Mineralogy, Sulfide minerals, Aqua regia, SEM, EDS spectra, Sulfur analysis

## Abstract

The objective of this study was to investigate the use of the acid production potential (AP) calculation factor and seven different S analysis methods in the preliminary mine waste characterization by analyzing and comparing 48 Finnish mine waste samples. Special attention was paid on mineralogical aspects and data produced in the exploration phase of a mining project.

According to our results, the abundance of sulfide species other than pyrite in Finnish mine waste suggests that the factor to calculate the AP should be considered based on mineralogy and would often be below 31.25. Therefore, the mineralogy-based determination of S should be preferred. However, the determination of S based on scanning electron microscope (SEM) mineralogy includes some uncertainties. Underestimation of S content may appear if not all S-bearing mineral particles have been detected, or if the amount of S is low in general. This uncertainty appears to be especially related to the samples containing elevated (> 9 wt%) amounts of serpentine, diopside, augite, and/or hornblende. Risk of overestimating AP is related to samples containing high amounts (> 4.13 wt%) of S-bearing minerals. These uncertainties can be reduced by inspecting that the SEM mineralogy-based S concentrations are in line with the energy dispersive X-ray spectrometer data. The aqua regia extractable S concentrations, which are often available in the exploration phase, appeared to be usable in the preliminary waste rock AP assessment and often comparable with the analytical total S values in the Finnish waste rock samples, especially when the samples did not contain any sulfate minerals. In contrast, the analytical sulfide S and the X-ray fluorescence methods may lead to an underestimation of AP.

## Introduction

Acid mine drainage (AMD) with elevated concentrations of harmful elements is one of the main concerns related to mine waste management (Dold, 2014; Lottermoser, 2010; MEND, 1991; Price, 2003). AMD is mainly related to the ore deposits containing Fe-sulfide minerals pyrite (FeS_2_) and pyrrhotite (Fe_1-x_S), which are prone to produce acid under the oxidating influence of atmospheric conditions (Blowes & Ptacek, 1994; Lottermoser, 2010; Singer & Stumm, 1970). The prediction of AMD generation is an essential part of the preliminary mine waste characterization (Dold, 2017; Parbhakar-Fox & Lottermoser, 2015). Mine waste, including waste rocks and tailings, should be characterized in an early phase of a mining project to investigate potential utilization possibilities of the waste and to design appropriate mine waste facilities and water treatment techniques (Kauppila et al., 2018). In general, management of mine waste should be included in the life-of-mine management models. Data for preliminary mine waste characterization and AMD prediction can be obtained already during the exploration phase of a mining project through geochemical and mineralogical analysis of drill cores penetrating future waste rock or from tailing samples produced in the processing tests. In general, it would be beneficial if the same methods that provide data for exploration could also be used in assessing the AMD generation potential of future mine waste.

Suitable methods to predict AMD generation include static and kinetic tests (Lapakko, 2002; Lottermoser, 2010). Static tests are short-term low-cost laboratory tests, commonly used for the preliminary screening, and to select suitable samples for further testing. Kinetic tests are longer-term, more expensive, and need case-specific planning and data from the preliminary tests, and are therefore not suitable for the preliminary screening. One of the most used static AMD prediction tests is the acid–base accounting (ABA) test, which includes the determination of the acid potential (AP) and the neutralization potential (NP) of the mine waste (Lapakko, 2002; Price, 2009; Sobek et al., 1978; White et al., 1999).

For the AP calculation, the sulfur concentration is the key parameter. Various methods exist for the determination of S concentration from the mine waste samples, and they can be divided into mineralogical analyses and geochemical determinations of total S or S species (CEN, 2012; Lapakko, 2002; Price, 2009; Punkkinen et al., 2021). The standard ABA method SFS-EN 15875 (SFS-EN, 2012) includes calculation of AP based on the total S, which is determined by high temperature (at least 1150 °C) combustion, e.g., ISO 15178 (ISO, 2000). According to the ABA standard, sulfidic S determination may be used aside from the total S analyses in the AP calculation. One of the sulfidic S analysis methods listed in the standard includes similar technique as in ISO 15178, but with a lower combustion temperature of around 800 °C (SFS-EN, 2012). However, no CEN or ISO standards exist for the analysis of sulfidic sulfur or other sulfur species in extractive waste (SFS-EN, 2012). Although several laboratory methods exist to analyze the amounts of different sulfur species (Ackerman et al., 2012; Lapakko, 2002; Mahanta et al., 2017; Okai et al., 2007; SFS-EN, 2012; Wilson et al., 1963; Yin & Catalan, 2003), these are more complicated and time-consuming, and therefore less suitable for a preliminary screening of large number of samples.

Several challenges have been identified related to the AP calculation and determination of S by the geochemical methods (Dold, 2017; Lottermoser, 2010; Parbhakar-Fox & Lottermoser, 2015). For example, in the standard SFS-EN 15875 (SFS-EN, 2012), the AP is calculated by multiplying the total S content by 31.25 (ratio of molecular masses of CaCO_3_ 100 g/mol and sulfur 32 g/mol) since it is assumed that most of the S is pyritic, and thus, four moles of protons (H^+^) are produced during the oxidation process via oxygen of one mole of pyrite (Colmer & Hinkle, 1947; Dold, 2014; Nordstrom, 2000; Singer & Stumm, 1970). However, some minerals, including non-acid producing barite (BaSO_4_), and acid-producing members of the alunite (KAl_3_(SO_4_)_2_(OH)_6_) – jarosite (KFe_3_(SO_4_)_2_(OH)_6_) series, might cause uncertainty for the AP calculation (Lapakko, 2002). Furthermore, the amount of H^+^ produced by different sulfides varies; chalcopyrite (CuFeS_2_) and pyrrhotite oxidation via oxygen produce two moles of H^+^, and the factor should therefore be divided by two, while sphalerite ((Zn,Fe)S) and galena (PbS) oxidation produce zero moles of H^+^, and the factor is therefore zero (Dold, 2017). Although the common assumption is that pyrite is the most frequent sulfide mineral present in the mine waste (Lottermoser, 2010; Nicholson, 1994), it has been presented that pyrrhotite might be the most common sulfide mineral in the Finnish waste rock (Karlsson et al., 2018). To tackle the problems related to the geochemical S analyses, mineralogy-based approaches have been proposed, where S concentrations and AP values are calculated for each mineral based on the modal mineralogy of the samples (Dold, 2017; Jamieson et al., 2015; Karlsson et al., 2018; Lawrence & Scheske, 1997; Parbhakar-Fox & Lottermoser, 2015).

The common geochemical exploration methods that could also be used to measure S include e.g., aqua regia (AR) extraction and X-ray fluorescence (XRF) analysis. AR, a leach utilizing a 3:1 mixture of hydrochloric acid and nitric acid (Doležal et al., 1968; Niskavaara, 1995), dissolves elements bound especially to the sulfide fraction, but it also decomposes all carbonates and secondary minerals, and partly some silicates. The XRF method (Criss & Birks, 1968) is widely used for the determination of the major constituents of rocks and it is also capable of providing useful data for many trace elements (Fletcher, 1981). Although the ISO 15178 (ISO, 2000) total S method has been demonstrated to be more accurate than the AR and XRF methods (Ackerman et al., 2012; Norrish & Thompson, 1990; Okai et al., 2007), and these methods have the same uncertainties as the other geochemical S analysis methods mentioned above, the usability of AR and XRF derived S concentrations in the preliminary AP assessment should be investigated, as in some cases, this data is abundant in the exploration phase and the use of it would decrease the need for high amount of additional analyses.

The objectives of this study included the assessment of the frequency of different S-bearing minerals in the Finnish mine waste, to assess if the general use of the AP factor of 31.25 is justified, and the suitability of seven different S analysis methods for the preliminary characterization of mine waste. Special attention was paid on the mineralogical aspects and data produced in the exploration phase of a mining project. In this study, 48 Finnish mine waste samples from 16 mine sites were analyzed. The seven investigated methods to determine the total amount of S or sulfidic S included (1) the analytical total S determination based on ISO 15178 standard, (2) analytical sulfidic S determination similar to the ISO 15178 but at lower combustion temperature (810 °C), (3) total S and (4) sulfidic S concentrations calculated based on the modal mineralogy investigated by field emission scanning electron microscope with an automated energy dispersive X-ray spectrometer (FE-SEM-EDS), and (5) the total S determined by EDS spectral data. The studied methods commonly used in the exploration phase of a mining project included (6) the AR extraction and (7) the XRF analysis.

## Materials and methods

For this study, 48 mine waste samples (45 waste rocks, 3 tailings) from 16 Finnish mine sites were investigated. The mine sites represent Archaean and Paleoproterozoic deposits, the commodities including Au, Co, Cr, Cu, Ni, P, Pb, platinum group elements, talc, and Zn. Of the 45 waste rock pile surface samples 30 were collected as 15–20 kg composite samples, and 15 as a single piece of rock.

The samples were pretreated and analyzed in a laboratory of Labtium Oy/Eurofins Labtium Oy, which has been accredited by FINAS (Finnish Accreditation Service) in accordance with the ISO/IEC 17025 standard (ISO/IEC, 2005). The rock samples were first dried at < 40 °C and then crushed (> 70% < 2 mm). The tailing samples were freeze dried. For the laboratory analyses, the samples were split using a riffle splitter and/or the cone and quartering method and milled in a steel container. The investigated methods to determine the total amount of S or sulfidic S are presented in Table 1 and described in more detail below. Table 2 presents which analytical methods were performed for which mine waste samples.

**Table 1:** The investigated methods to determine the total amount of S or sulfidic S. Geochemical methods include the analytical total S and sulfidic S, aqua regia (AR) extraction, and X-ray fluorescence (XRF) method. Mineralogical methods include the calculation of total S and sulfidic S based on scanning electron microscope (SEM) modal mineralogy, and total S based on the energy dispersive X-ray spectrometer (EDS) sum spectra. *n.a.*, not available

**Method**	**Standard**	**Reference**
Analytical total S	ISO 15178	(ISO, 2000)
Analytical sulfidic S	n.a	(SFS-EN, 2012)
Aqua regia (AR) extraction	ISO-11466	(ISO, 1995)
Total S by X-ray fluorescence analysis (XRF)	n.a	(Criss & Birks, 1968)
Total S based on SEM modal mineralogy	n.a	(Dold, 2017; Karlsson et al., 2018)
Sulfidic S based on SEM modal mineralogy	n.a	(Dold, 2017; Karlsson et al., 2018)
Total S based on EDS sum spectra	n.a	n.a

**Table 2:** List of analytical methods performed on the mine waste and standard samples. *WR*, waste rock; *FE-SEM-EDS*, field emission scanning electron microscope with an automated energy dispersive X-ray spectrometer; *AR*, aqua regia extraction method; *XRF*, X-ray fluorescence method; *n.a*., not available. In addition, samples 1–5, 7–8, 10–12, 15–17, 27, and 32 were analyzed with the NH_4_-acetate extraction and NH_4_-oxalate methods

**Sample #**	**Sample type**	**Min. analysis**	**Min. S calculations**	**EDS Sum.sp. S**	**Analytical Tot S**	**Analytical Sulf S**	**AR S**	**XRF S**
1–30	WR composite	FE-SEM-EDS	X	X	X	X	X	X
31	WR composite	FE-SEM-EDS	X	n.a	X	X	X	n.a
32–34	Tailings	FE-SEM-EDS	X	X	X	X	X	X
35–48	WR single rock	Light micr	n.a	n.a	X	X	X	X
QC1-2	Standard	n.a	n.a	n.a	X	n.a	n.a	n.a
QC3-4	Standard	n.a	n.a	n.a	n.a	X	n.a	n.a
QC5-6	Standard	n.a	n.a	n.a	n.a	n.a	X	n.a
QC7-8	Standard	n.a	n.a	n.a	n.a	n.a	n.a	X

The analytical total concentrations of S were determined according to the ISO 15178 (ISO, 2000) standard, which is based on combustion of a sample in O_2_ flow at 1400 °C, and the measurement of oxidized SO_3_ with infrared detector. The equipment consisted of CS 580 carbon/sulfur analyzer (Eltra GmbH). The combustion time varies from 1.5 up to 7 min, depending on the sample material. Same equipment as for the analytical total S concentration was used to determine the analytical sulfidic S concentration, but the combustion temperature was lower, 810 °C, and the combustion time 3.5 min (see SFS-EN, 2012). The AR extraction was performed based on a modification of the ISO-11466 standard (ISO, 1995), and the AR extracted S contents were determined with ICP-OES technique using the standard SFS-EN ISO 11885 (SFS-EN, 2007) and iCAP 6500 Duo equipment (Thermo Electron). The total concentrations of S were also determined by the X-ray fluorescence (XRF) method (Criss & Birks, 1968) utilizing AXIOS equipment (PANalytical).

To investigate the occurrence of secondary and readily leachable S minerals that might be left undetected in the mineralogical investigation, the samples 1–5, 7–8, 10–12, 15–17, 27, and 32 were parallelly analyzed with the NH_4_-acetate extraction and NH_4_-oxalate extraction methods, utilizing the ICP-OES technique. These methods were not included in the total amount of S or sulfidic S determination methods listed in Table 1 but were included in the study to assess the amount of S bound to secondary minerals that might affect the other S analysis methods. The NH_4_-acetate extraction liberates the exchangeable fraction consisting of chemically adsorbed elements and phases bound to carbonates, gypsum, and other salts, and included 1 M NH_4_-acetate leach at pH4.5 with a solid:solution ratio of 1:60, shaken for 2 h at room temperature (see Dold, 2003; Heikkinen & Räisänen, 2008). The NH_4_-oxalate extraction liberates phases bound to poorly crystalline Fe oxyhydroxides and secondary jarosite, and partly elements bound to crystalline Fe and Mn oxyhydroxides and oxides (see Dold, 2003; Heikkinen & Räisänen, 2008). It included a 0.2 M NH_4_-oxalate leach at pH3.0 with a solid:solution ratio of 1:100, shaken for 4 h at room temperature in darkness.

The quality of the geochemical analysis was assured by the accredited laboratory by analyzing standard and blind control samples and providing the quality control analysis results along with the sample results. Used quality control standard samples included QC1 (GS310-7 by Geostats PTY Ltd.), QC2 (GS900-5 by Geostats PTY Ltd.), QC3 (Eurofins Labtium in-lab standard), QC4 (Eurofins Labtium in-lab standard), QC5 (TILL-2, by Canmet), QC6 (TILL-4 by Canmet), QC7 (GBMS304-6 by Geostats PTY Ltd.), and QC8 (Eurofins Labtium in-lab standard). For the geochemical data analysis, detector readings below the detection limit (DL) were replaced by a value of 0.

Mineralogical analyses were conducted at the Geological Survey of Finland (GTK). Samples 1–36 were analyzed by FE-SEM-EDS at the GTK Research Laboratory, which follows Quality Management System Standard ISO 9001 (DNV-GL). The equipment consisted of JEOL JSM7100F Schottky, combined with an Oxford Instruments EDS spectrometer X-Max 80 mm^2^ (SDD). The powdered mine waste samples prepared for the geochemical analyses were also used to prepare mineralogical polished samples cast in epoxy and covered with graphite to enhance electrical conductivity. The modal mineralogy was investigated by analyzing around 10,000 individual mineral particles from each sample utilizing the INCA Feature software (version 5.05 by Oxford Instruments). The sum spectra were generated over the sample preparation surfaces by montage technique with Aztec software (version 3.4 by Oxford Instruments). The results are semi-quantitative and were normalized to 100%.

Minerals were identified by comparing the numerical element composition obtained from EDS spectra with the mineralogical database of GTK. For technical reasons, around 5–10% of mineral identifications are usually classed as “unclassified.” This class mainly includes mixed analyses generated from various mineral phases. The amount of unclassified minerals is usually larger when analyzing fine particle samples or samples with complex mineralogy. In this study, the “unclassified” phase was normalized by dividing it for the identified mineral phases based on the identified mineral amounts.

Precise identification of minerals based on EDS spectra is not always possible, for instance because carbon and OH and H_2_O groups are not shown in the analysis, which should be considered when examining the results. The accuracy of mineral classification based on the GTK mineral database was routinely monitored for each analyzed sample. The analytical data that were left unclassified were checked manually.

The mineralogical S calculations were based on the modal mineralogy of the samples and mineral formulas (Dold, 2017; Karlsson et al., 2018). Mineralogical total S of a sample was calculated by multiplying the wt% of S in the detected mineral with the wt% of the mineral in the sample and summing up the S of all the detected S containing minerals. The mineralogical sulfidic S was calculated accordingly, but only sulfide minerals were considered. The total S based on the EDS sum spectra was obtained straight from the data. Samples 35–48 were analyzed by light microscopy (petrographic microscope) and were not included in the mineralogical total and sulfidic S calculations.

## Results

The contents and the amount of the S-bearing minerals were variable in the investigated samples (Table 3). However, the most common sulfide minerals were the iron sulfides, pyrrhotite, and pyrite, whereas the As-sulfides were the least common. Pyrrhotite was detected in 31 out of 34 samples analyzed with SEM and its average content was 1.48 wt% (min 0.01 wt%, max 8.34 wt%), while pyrite was detected in 23 out of 34 samples, with an average content of 0.77 wt% (min 0.01, max 9.97 wt%) indicating that pyrrhotite is typically the most common sulfide mineral in Finnish waste rocks. The As-sulfides, gersdorffite (NiAsS) and arsenopyrite (FeAsS), occurred both only in one sample (average content 0.01 wt% and 0.03 wt%, respectively). Of the base metal sulfides, chalcopyrite was the most common followed in abundance by the pentlandite ((Fe,Ni)_9_S_8_) and sphalerite. Chalcopyrite was detected in 20 out of 34 samples, with an average content of 0.15 wt% (min 0.01 wt%, max 0.90 wt%), pentlandite in 15 out of 34 samples (average content 0.25 wt%, min 0.01 wt%, max 2.09 wt%), and sphalerite in 6 out of 34 samples (average content 0.16 wt%, min 0.01 wt%, max 0.78 wt%). A secondary mineral phase classified as oxidized Fe-sulfides was quite common and it was detected in 16 out of 34 samples (average content 0.25 wt%, min 0.01 wt%, max 1.04 wt%). According to the FE-SEM-EDS data, the average composition of the oxidized Fe-sulfide class was O 22 wt%, Fe 44 wt% and S 32 wt%. Other detected S minerals included gypsum (CaSO_4_·2H_2_O), Fe-sulfate (FeSO_4_), and barite in the order of abundance. Gypsum was detected in 9 out of 34 samples (average content 0.01 wt%, min 0.01 wt%, max 0.03 wt%), Fe-sulfate in 3 out of 34 samples (average content 0.04 wt%, min 0.03 wt%, max 0.04 wt%), and barite in only two samples (0.6 wt% and 77.48 wt%).

**Table 3:** Analytical results of the investigated mine waste samples (tailings marked with “*”, other samples are waste rocks). Values as wt%. *Min tot S*, total S calculated by mineralogy; *Min Sulf S*, sulfidic S calculated by mineralogy; *Sum sp. S*, S detected by the EDS sum spectra; *Analytical Tot S*, total S analyzed by the ISO 15178 standard; *Analytical Sulf S*, sulfidic S determination similar to ISO 15178 but at 810 °C; *AR S*, aqua regia extractable S; *XRF S*, S detected by the X-ray fluorescence method; *NH*_*4*_*-ac*. *S*, NH_4_-acetate extractable S; *NH*_*4*_*-ox. S*, NH_4_-oxalate extractable S. The average S contents are calculated for samples 1–34, excluding the sample 31 for which no Sum sp. S nor XRF S data was available. *n.a*., not available. The quality control samples QC1-8 with ± standard deviation

**#**	**Main minerals**	**Sulfides**	**Other S minerals**	**Min Tot S**	**Min Sulf S**	**Sum sp. S**	**Analytical Tot S**	**Analytical Sulf S**	**AR S**	**XRF S**	**NH**_**4**_**-ac. S**	**NH**_**4**_**-ox. S**
1	srp 78, ol 11	po 0.15, pe 0.06, ch 0.03	ox-fe 0.07	0.11	0.09	0.65	0.63	0.12	0.57	0.55	0.12	0.13
2	di 42, am 29, srp 9	po 0.15, py 0.05, pe 0.04, ch 0.02	gy 0.01	0.11	0.11	0.77	0.29	0.02	0.30	0.27	0.004	0.005
3	di 41, am 26, srp 12, hbl 7	po 0.26, pe 0.06, ch 0.02	gy 0.01	0.13	0.13	0.72	0.21	< 0.01	0.22	0.20	0.003	0.004
4	srp 74, tlc 14	po 0.02, pe 0.01	ox-fe 0.02	0.02	0.01	0.75	0.14	< 0.01	0.15	0.15	0.04	0.05
5	qtz 25, pl 23, bt 16, am 8, ms 6	po 1.53, py 0.97, pe 0.19, ch 0.16	ox-fe 0.23	1.31	1.24	2.76	2.28	1.93	2.44	2.24	0.10	0.17
6	qtz 32, pl 27, bt 26, chl 5, ms 5	po 0.45, ch 0.04	fe-su 0.03	0.20	0.19	0.40	0.43	0.30	0.40	0.48	n.a	n.a
7	qtz 24, ms 21, bt 21, pl 14	po 6.57, pe 0.29, ch 0.24, py 0.15, sph 0.02	ox-fe 0.67	3.05	2.84	4.75	5.32	4.76	4.38	5.54	0.05	0.07
8	pl 37, bt 27, qtz 15, ms 8	po 4.45, py 0.11, ch 0.07, sph 0.01	ox-fe 0.25	1.91	1.83	2.59	2.47	2.21	2.22	2.37	0.07	0.08
9	qtz 29, bt 27, pl 22, bt 14	po 1.97, py 0.26	ox-fe 0.18	0.97	0.91	1.35	1.35	1.38	1.28	1.32	n.a	n.a
10	bt 36, pl 23, qtz 18, hbl 7	po 3.02, pe 0.03, py 0.02	ox-fe 0.15	1.25	1.20	1.77	1.69	1.66	1.57	1.57	0.03	0.04
11	bt 37, pl 22, qtz 15, chl 8	po 2.28, py 0.41, ch 0.04, pe 0.01	ox-fe 0.32	1.23	1.13	1.57	1.85	1.41	1.55	1.52	0.08	0.12
12	bt 28, pl 18, qtz 10, chl 10, am 8	po 2.60, py 0.70, ch 0.01	ox-fe 0.11	1.43	1.39	1.31	1.71	1.33	1.43	1.30	0.04	0.05
13	pl 38, aug 37, hbl 20	ch 0.33, po 0.12		0.16	0.16	0.47	0.51	0.34	0.46	0.51	n.a	n.a
14	aug 35, pl 33, hbl 15, bt 10	ch 0.12, py 0.03	gy 0.02	0.06	0.06	0.34	0.52	0.15	0.26	0.28	n.a	n.a
15	pl 48, hbl 23, aug 11, qtz 5	ch 0.17, po 0.12, py 0.01		0.11	0.11	0.29	0.37	0.24	0.33	0.34	0.004	0.006
16	pl 33, qtz 21, kfs, 14, bt 11, hbl 9, am 8	po 0.10		0.04	0.04	0.11	0.10	0.05	0.10	0.12	0.003	0.007
17	di 51, am 19, srp 10, hbl 5	po 0.16, pe 0.05, ch 0.03		0.09	0.09	0.34	0.31	0.05	0.28	0.30	0.007	0.009
18	pl 31, bt 20, kfs 16, qtz 12	ch 0.03, po 0.01		0.01	0.01	0.08	0.09	0.02	0.10	0.08	n.a	n.a
19	bt 34, cc 19, 14, phl 8, dol 7	po 0.01		0.00	0.00	0.08	0.07	< 0.01	0.10	0.07	n.a	n.a
20	pl 46, kfs 16, bt 15, aug 15, cc 5	py 0.22, po 0.12	ox-fe 0.01	0.17	0.16	0.22	0.17	0.02	0.20	0.17	n.a	n.a
21	qtz 47, bt 18, pl 17	po 2.55, sph 0.78, py 0.78, pe 0.02	ox-fe 0.12, gy 0.01	1.72	1.68	1.35	1.65	1.03	1.59	1.32	n.a	n.a
22	qtz 41, chl 13, pl 10, mgs 10, tlv 7, am 5	po 1.19, py 0.82, pe 0.11, ge 0.01	ox-fe 0.08, gy 0.01	0.97	0.94	2.38	1.41	0.65	1.32	1.12	n.a	n.a
23	qtz 46, bt 11, mgs 10, pl 8, am 7, tlc 5	po 3.69, py 0.65, pe 0.61	ox-fe 0.04	2.01	1.99	1.81	2.27	1.52	2.27	1.84	n.a	n.a
24	pl 32, qtz 19, bt 17, py 10, phl 8, am 6, ms 5	py 9.97, po 0.69, sph 0.10, pe 0.05	ox-fe 0.02, gy 0.01	5.66	5.65	4.44	4.32	3.32	4.06	3.21	n.a	n.a
25	qtz 23, bt 22, pl 18, srp 11, po 8, ms 6	po 8.34, py 1.23, ch 0.90, sph 0.01	ox-fe 1.04	4.57	4.24	2.88	3.35	2.59	3.08	2.71	n.a	n.a
26	qtz 53, pl 11, chl 9, bt 9, ms 5	po 1.03, ch 0.10, py 0.07	ox-fe 0.61, gy 0.02	0.67	0.48	2.93	1.62	1.16	1.46	1.40	n.a	n.a
27	hbl 54, ol 22, di 7	pe 2.09, po 1.58, ch 0.46, py 0.04	fe-su 0.04	1.50	1.49	1.01	1.19	0.58	1.06	0.84	0.008	0.01
28	pl 43, qtz 22, bt 12, chl 6	po 0.8, py 0.6		0.63	0.63	1.13	1.26	0.47	1.17	0.73	n.a	n.a
29	pl 32, qtz 29, chl 19, bt 9	po 1.1, py 0.3	ox-fe 0.1	0.59	0.59	0.86	0.92	0.18	0.82	0.58	n.a	n.a
30	pl 35, qtz 23, bt 14, hbl 10, chl 10	po 0.58, py 0.13		0.30	0.30	0.76	0.82	0.14	0.76	0.56	n.a	n.a
31	ba 77, dol 9	py 0.10, sph 0.04	ba 77.48	10.71	0.07	n.a	10.40	0.04	0.19	n.a	n.a	n.a
32*	hbl 64, ol 13, di 9, am 7	po 0.26, pe 0.09, ch 0.03	fe-su 0.04, gy 0.01	0.15	0.14	0.73	0.72	0.32	0.64	0.55	0.02	0.02
33*	pl 37, qtz 18, kfs 14, bt 10, am 7, hbl 6	ch 0.04, py 0.03, ars 0.03	gy 0.03	0.04	0.03	0.18	0.06	0.02	0.05	0.23	n.a	n.a
34*	am 60, di 20, srp 9	po 0.10, ch 0.10		0.08	0.08	0.27	0.29	0.08	0.27	0.37	n.a	n.a
			**Average**	**0.95**	**0.91**	**1.27**	**1.22**	**0.85**	**1.12**	**1.06**		
35	qtz 90, ky 5–10	n.a	n.a	n.a	n.a	n.a	0.44	0.28	0.40	0.37	n.a	n.a
36	srp 90, ol 5	n.a	n.a	n.a	n.a	n.a	1.00	0.03	0.85	0.69	n.a	n.a
37	pl 47, bt 21, qtz 20	n.a	n.a	n.a	n.a	n.a	1.68	1.45	1.48	1.35	n.a	n.a
38	pl 35, am 34, qtz 19, bt 8	n.a	n.a	n.a	n.a	n.a	0.42	0.24	0.37	0.33	n.a	n.a
39	grt 50, px 40, pl 10	n.a	n.a	n.a	n.a	n.a	0.05	0.01	0.04	0.05	n.a	n.a
40	ky 50, qtz 30, crd 14	n.a	n.a	n.a	n.a	n.a	4.60	3.43	4.34	3.89	n.a	n.a
41	tlc, ms	n.a	n.a	n.a	n.a	n.a	0.56	0.40	0.50	0.44	n.a	n.a
42	tlc 66, carb 23, crm 11	n.a	n.a	n.a	n.a	n.a	0.08	0.01	0.06	0.05	n.a	n.a
43	tlc 66, carb 23, crm 11	n.a	n.a	n.a	n.a	n.a	0.04	< 0.01	0.05	0.03	n.a	n.a
44	qtz 50, bt 25, pl + kfs 15, mgt	n.a	n.a	n.a	n.a	n.a	0.11	0.02	0.10	0.09	n.a	n.a
45	tlc 71, carb 25	n.a	n.a	n.a	n.a	n.a	0.05	< 0.01	0.05	0.04	n.a	n.a
46	gr + po 60, qtz 25, bt 10, pl 5	n.a	n.a	n.a	n.a	n.a	6.36	5.66	5.34	4.80	n.a	n.a
47	carb 51, tlc 48	n.a	n.a	n.a	n.a	n.a	0.61	0.19	0.58	0.39	n.a	n.a
48	ky, am/px	n.a	n.a	n.a	n.a	n.a	0.52	0.27	0.48	0.34	n.a	n.a
QC1	Expected S 10.92, *n* = 7	n.a	n.a	n.a	n.a	n.a	10.77 ± 0.16	n.a	n.a	n.a	n.a	n.a
QC2	Expected S 0.34, *n* = 6	n.a	n.a	n.a	n.a	n.a	0.35 ± 0.01	n.a	n.a	n.a	n.a	n.a
QC3	Expected S 1.39, *n* = 6	n.a	n.a	n.a	n.a	n.a	n.a	1.06 ± 0.03	n.a	n.a	n.a	n.a
QC4	Expected S 6.54, *n* = 2	n.a	n.a	n.a	n.a	n.a	n.a	5.04 ± 0.08	n.a	n.a	n.a	n.a
QC5	Expected S < 0.05, *n* = 2	n.a	n.a	n.a	n.a	n.a	n.a	n.a	0.03 ± 0.0001	n.a	n.a	n.a
QC6	Expected S 0.08, *n* = 11	n.a	n.a	n.a	n.a	n.a	n.a	n.a	0.07 ± 0.003	n.a	n.a	n.a
QC7	Expected S 2.01, *n* = 9	n.a	n.a	n.a	n.a	n.a	n.a	n.a	n.a	1.75 ± 0.17	n.a	n.a
QC8	Expected S 0.10, *n* = 9	n.a	n.a	n.a	n.a	n.a	n.a	n.a	n.a	0.09 ± 0.02	n.a	n.a

*am* amphibole (actinolite, anthophyllite, cummingtonite, tremolite), *ap* apatite, *ars* arsenopyrite, *aug* augite, *bt* biotite, *carb* undefined carbonate, *cc* calcite, *ch* chalcopyrite, *chl* chlorite, *crd* cordierite, *crm* chromite, *crs* chrysotile, *di* diopside, *dol* dolomite, *ep* epidote, *fe-su* Fe-sulfate, *ge* gersdorffite, *gö* göthite, *gr* graphite, *grt* garnet, *gy* gypsum, *hbl* hornblende, *ilm* ilmenite, *ja* jarosite, *kfs* k-feldspar, *ky* kyanite, *li* limonite, *mgs* magnesite, *mgt* magnetite, *ms* muscovite, *ol* olivine, *ox*-*fe* oxidized Fe-sulfide, *pe* pentlandite, *phl* phlogobite, *pl* plagioclase, *po* pyrrhotite, *px* pyroxene, *py* pyrite, *qtz* quartz, *sph* sphalerite, *srp* serpentine, *tlc* talc

Some minor deviation could be observed between the amounts of secondary S minerals (oxidized Fe-sulfide, Fe-sulfate, and gypsum) detected in modal mineralogy and the amounts of S leached by the NH_4_-acetate and NH_4_-oxalate extractions made for the 15 selected samples (Table 3). The S bound to these secondary S minerals is presented by the difference between Min Tot S and Min Sulf S in Table 3, excluding the barite-bearing sample 31. For samples 15–17, no S-bearing minerals besides sulfides were detected by modal mineralogy; however, some minor amounts of readily leachable S were detected by NH_4_-acetate (0.004, 0.003, and 0.007 wt%, respectively) and NH_4_-oxalate (0.006, 0.007, and 0.009 wt%, respectively) extractions. For samples 2, 3, 8, 10–12, 27, and 32, the amounts of detected secondary S minerals and readily leachable S were close to each other, in general the leachable amounts being slightly (up to 0.01 wt%) higher. For samples 1, 4, and 5, the amounts of readily leachable S were up to 0.11 wt% higher than the amount of S bound to the detected secondary S minerals. For sample 7, the amount of S bound to the detected secondary S-bearing mineral-oxidized Fe-sulfide was higher (0.21 wt%) than the NH_4_-oxalate extractable S (0.07 wt%).

The content of S showed marked variation depending on the analytical method (Table 3). Of the geochemical analyses, the analytical total S resulted in the highest average values, followed by AR S and XRF S, while the smallest values were measured by the analytical sulfidic S method (samples 1–30 and 32–34 included in the average). The average analytical total S was 1.22 wt%, while the average analytical sulfidic S was 0.85 wt%. The average AR extractable S was 1.12 wt%, and the average total S detected by XRF 1.06 wt%.

The mineralogy-based calculations for the S showed lower S content than the analytical methods except for the total S calculated from the EDS sum spectra, which was the highest of all the S analyses and as such, also higher than the average total S and sulfidic S calculated based on the modal mineralogy for the samples analyzed with SEM (excluding the high-barite sample 31 without EDS sum spectra). The average for the EDS sum spectra S was 1.27 wt%, while the average total S based on the modal mineralogy was 0.95 wt%, and the average mineralogy-based sulfidic S 0.91 wt%.

Table 3 shows that there is a clear difference between the total S based on SEM modal mineralogy and the analytical total S. The total S based on SEM modal mineralogy was higher than the analytical total S for five samples (21, 24, 25, 25, and 31) out of 34. In these cases, the total amount of S-bearing minerals was high (> 4.13 wt%), and the average analytical total S was 4.18 wt%. The total S based on SEM modal mineralogy was > 50% lower than the analytical total S for 16 samples (1–2, 4, 6, 13–19, 26, 28, 30, 32, and 34). In these cases, the average analytical total S was 0.51 wt%. These samples often contained serpentine ((Mg,Fe)_3_Si_2_O_5_(OH)_4_), diopside (CaMgSi_2_O_6_), augite ((Ca,Na)(Mg,Fe,Al,Ti)(Si,Al)_2_O_6_), and hornblende ((Ca,Na)_2–3_(Mg,Fe,Al)_5_(Al,Si)_8_O_22_(OH,F)_2_). For the rest of the samples investigated by the SEM mineralogical method (3, 5, 7–12, 20, 22–23, and 29), the total S based on SEM modal mineralogy was < 50% lower than the analytical total S, and the average analytical total S was 1.67 wt%.

**Fig. 1:**
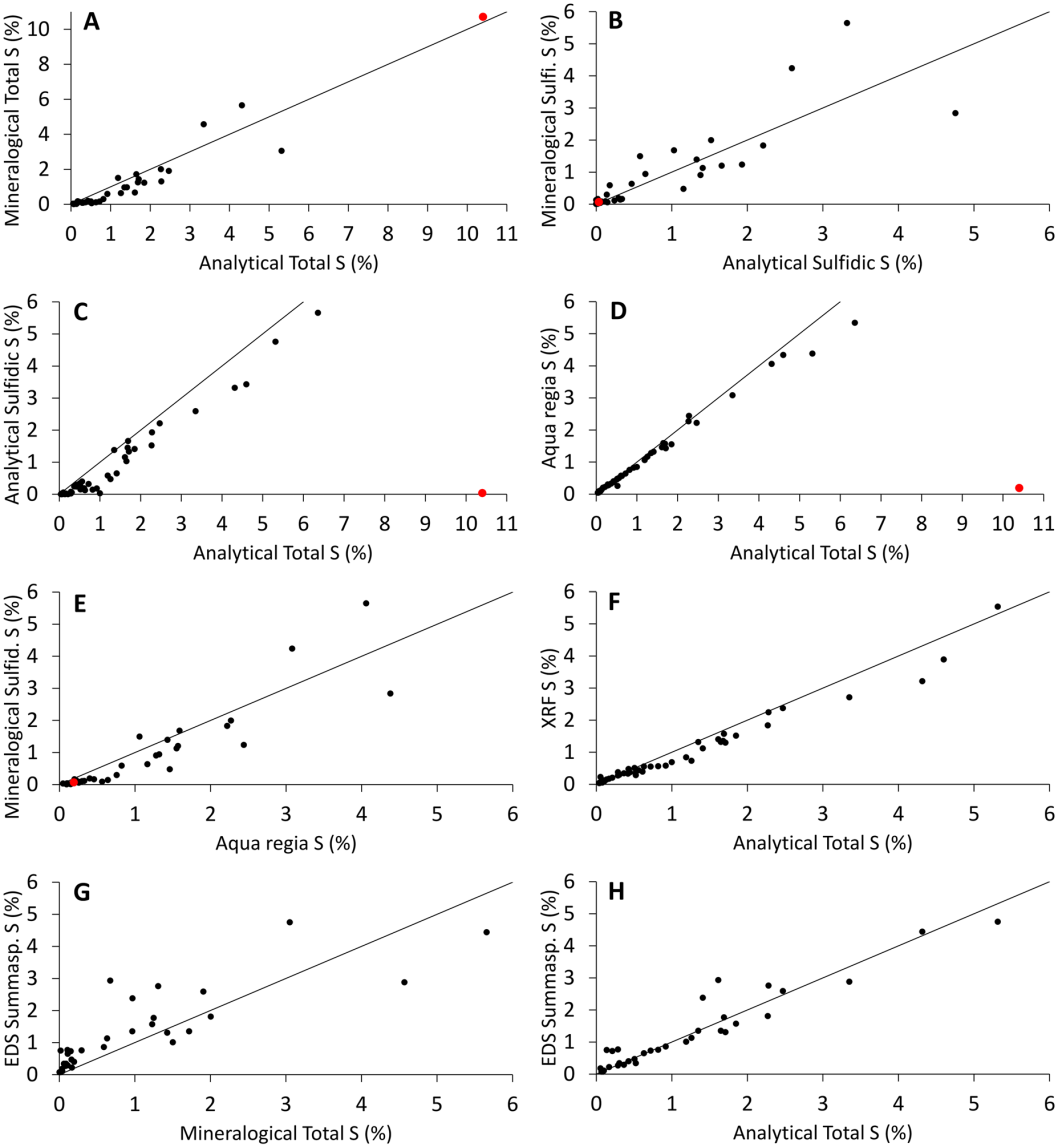
Comparison of S concentrations based on different S analysis methods. The red dots represent the high-baryte (~ 10 wt% barytic S) and low-sulfide sample (number 31). Equation of the lines is *y* = *x*, which have been added for reference and do not reflect the analysis results

Comparison of the S content of each sample based on the different methods is presented in Fig. 1. In general, the analytical total S was higher than the total S calculated based on SEM modal mineralogy, analytical sulfidic S, and XRF S (Fig. 1A, C, and F, respectively). It was nevertheless at the same level as the total S derived from the EDS sum spectra (Fig. 1H) and the AR extractable S, the latter especially when S wt% was < 3 (Fig. 1D). The total S derived from the EDS sum spectra was generally higher than the total S calculated by the SEM modal mineralogy (Fig. 1G), (Fig. 1H) with EDS sum spectra capturing more S than the modal mineralogical approach. However, some divergence existed in the values between the total S derived from the EDS sum spectra and the total S calculated by the SEM modal mineralogy (Fig. 1G). For example, samples 5, 7, 22, and 26 had notably higher EDS sum spectra S wt% compared with the total S calculated by SEM modal mineralogy (2.76 vs. 1.31, 4.75 vs. 3.05, 2.38 vs 0.97, and 2.93 vs. 0.67, respectively), whereas, for example, samples 24 and 25 had lower EDS sum spectra S than the total S based on SEM modal mineralogy (4.44 vs. 5.66 and 2.88 vs. 4.57, respectively). Sulfidic S values calculated based on the modal mineralogy were in general lower than the AR extractable S values (Fig. 1E) but at the same level as the analytical sulfidic S results on average, even though the results were not one-to-one (Fig. 1B).

The S concentrations between the different methods showed clear difference for the sample containing high amount of barite (sample 31). Whereas the analytical total S method and the total S calculation based on the modal mineralogy resulted in high S concentration for the sample (Fig. 1A, the red dot), the sulfidic S methods (analytical sulfidic S method, sulfidic S calculation based on modal mineralogy, and the AR extraction) resulted in close to 0% of S (Fig. 1B and D) suggesting that the latter methods do not analyze barite.

**Fig. 2:**
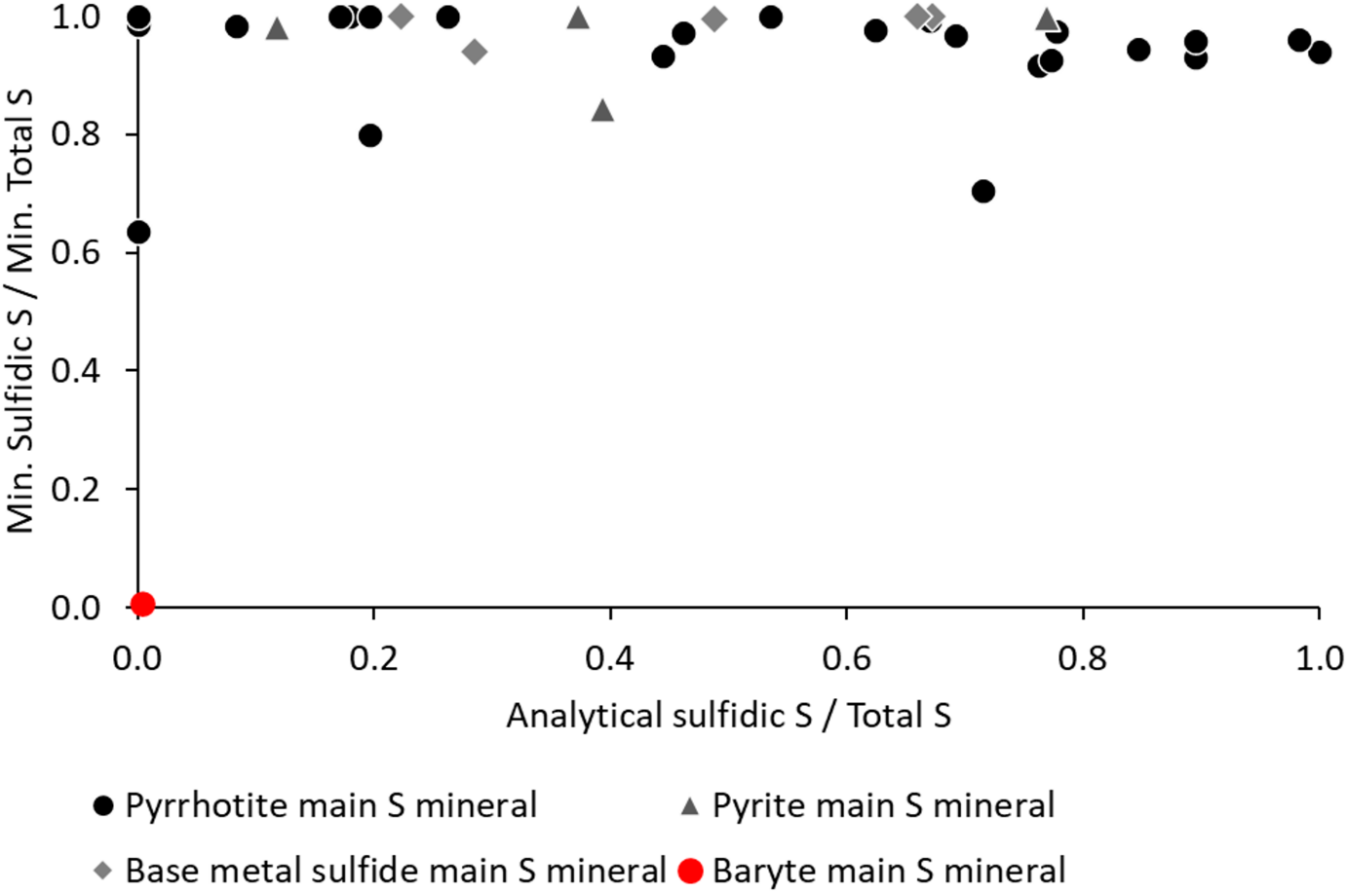
The ratio of sulfide S and total S calculated based on modal mineralogy (*y*-axis) versus the ratio of analytical sulfidic S and total S (*x*-axis)

The comparison of the ratios of sulfide S and the total S calculated based on the SEM mineralogy and the respective ratios of the analytical results did not show clear correlation (Fig. 2). In general, the mineralogy-based sulfidic S and total S ratios were high and close to one, while many of the analytical sulfidic S and total S ratios were lower than unity, suggesting that analytical sulfide S did not correspond to the amount of sulfides in the samples, or the mineralogical total S missed part of the S minerals in the samples. The species of the main sulfide mineral in the sample appeared not to affect this phenomenon significantly.

## Discussion

Our results from the 16 Finnish mines indicated that pyrrhotite is the most common sulfide mineral in the Finnish waste rock, followed by pyrite, as also previously presented by Karlsson et al. (2018), and thus accounts for most of the sulfidic S in the waste. The abundance of pyrrhotite is in contrast with the common assumption that pyrite is the most frequent sulfide mineral present in mine waste (Lottermoser, 2010; Nicholson, 1994). Of the base metal sulfides, chalcopyrite appears to be the most common, followed by pentlandite and sphalerite, but in general, they occur less frequently in the Finnish waste rock than the Fe-sulfides. Other sulfide minerals, including gersdorffite and arsenopyrite, which were detected at one mine site each, are the rarest based on our data.

The abundance of pyrrhotite suggests that the factor to calculate the AP by multiplying the S content should be considered based on the dominating sulfide species, as the amount of H^+^ released in the oxidation processes varies for different sulfides. For example, four moles of H^+^ is produced during the oxidation process of one mole of pyrite via oxygen, whereas pyrrhotite oxidation produces only two moles of H^+^ (Colmer & Hinkle, 1947; Dold, 2014, 2017; Nordstrom, 2000; Singer & Stumm, 1970). Therefore, in most of the Finnish mine waste cases, the AP calculation factor should be lower than the commonly used 31.25 which assumes that all S is pyritic. This further highlights the importance of preferring a mineralogy-based S determination method for the calculation of AP over the geochemical methods.

Other sulfur-bearing minerals, which might complicate the geochemical S analysis and the estimation of acid production potential of the mine waste material, include sulfates like alunite-jarosite series minerals and non-acid generating barite (Lapakko, 2002; Price, 2009). In particular, the weathered mine waste material might also contain secondary sulfate minerals, like gypsum or jarosite, and other S containing minerals classified as oxidized sulfides. In the investigated mine waste samples, the S-bearing minerals other than sulfides or oxidized Fe-sulfides were relatively rare (e.g., barite, detected in 1 out of 34 samples investigated by SEM) or occurred in small amounts (e.g., gypsum and Fe-sulfate ≤ 0.04 wt%, detected in 12 out of 34 samples investigated by SEM). Thus, their influence in the AP evaluation can be considered only minor in fresh Finnish waste rocks. However, barite is a relatively common mineral in the Finnish mineral deposits, occurring typically in the middle and low temperature hydrothermal veins and cavities of magmatic rocks (Hytönen, 1999), and its presence should always be investigated when characterizing mine waste materials to avoid the overestimation of AP. The oxidized Fe-sulfides, which were detected in half of the samples investigated by SEM, occurred also in relatively high concentrations (up to 1.04 wt% in sample 25), and should be considered when estimating the AP of weathered mine waste material. Based on the mineralogical data, excluding the barite-cases, the total S results mainly present sulfidic S amounts in fresh Finnish waste rocks. However, a geologist should always make a mineralogical assessment of the deposit under investigation, to identify the potential minerals affecting the AP calculation.

Comparison of the seven different analytical and mineralogy-based S methods showed that the EDS sum spectral S and the analytical total S methods resulted on average in the highest S values of all the methods and were often on the same level (Fig. 1H). However, this might lead to overestimation of AP in some cases when using total S in the AP calculations. The high temperature in the total S standard method combined with the presence of oxygen gas is assumed to transform all the sulfur species into sulfur dioxide (Price, 2009). The known downside of this commonly used standard method is the risk for overestimation of the AP potential if the sample contains also non-acid producing S-bearing minerals aside with the acid producing minerals (Dold, 2017; Karlsson et al., 2018). As demonstrated in this study (Fig. 1), the high temperature of the method also volatilizes barite, which causes an overestimation error in the AMD prediction when barite is present in the waste rock. However, as stated by Punkkinen et al. (2021), the analytical total S results are always “on the safe side” when assessing AMD potential; either correct or too high, but never too low.

In contrast to the analytical total S, the total S calculated by SEM modal mineralogy was on average lower than the other total S methods, i.e., the analytical total S and the EDS sum spectral S, which might lead to underestimation of AP. An explanation for the difference of the two mineralogical methods could be that when determining the SEM modal mineralogy, around > 3 µm particles are taken into account. In contrast, the EDS sum spectra is generated by rastering the beam over the entire measurement area with analytical spot size well below 1 µm (Oxford Instruments, 2021). Sulfides can occur even in below 1 µm particle sizes (Greer, 1978), which complicates their detection. Furthermore, they are relatively brittle minerals (Hytönen, 1999), which might lead to enrichment of sulfides into the smallest size fraction during the grinding of the sample and, as a result, these sulfides would not be measured in the SEM modal mineralogy method. In addition, secondary minerals formed by the alteration of sulfides are often fine-grained by nature, potentially forming another group of minerals being left unanalyzed. According to the NH_4_-acetate and NH_4_-oxalate extraction results (Table 3), the amount of readily leachable S is indeed often slightly higher than the amount of S bound to the secondary minerals detected in the sample, indicating that some secondary minerals remain undetected by the SEM mineralogical investigation. However, the relatively small amounts of S detected by the NH_4_-acetate and NH_4_-oxalate extractions are not sufficient to explain the differences between SEM modal mineralogy and EDS sum spectra. Another source of error in the modal method could be related to S being bound in trace element concentrations, i.e., below the limit of detection (LOD) of SEM, to certain mineral phases. It should also be highlighted that the quality of EDS data is semiquantitative. In addition, the sum spectra and the modal mineralogy measurement are not entirely comparable methodically, considering the material subjected to analysis. The sum spectra is generated over the entire or nearly entire surface area of the sample preparation, whereas the modal mineralogy measurement is based on 10,000 randomly analyzed mineral grains within the preparation. Thus, the modal mineralogy measurement leaves room for a potential nugget effect that is more pronounced in samples with low S-bearing mineral content.

The differences between the total S based on SEM modal mineralogy, and the total S based on EDS sum spectral and the analytical total S appear to be related to the mineralogical properties of the samples. Based on the results, if the amount of S-bearing minerals is high (in this study > 4.13 wt%), there is an elevated risk (but not certainty) that the total S based on SEM modal mineralogy is overestimated. The samples with high sulfide content or deviant element composition can lead to the overestimation of S based on modal mineralogy, because the calculations are based on the ideal average compositions of sulfides from the literature. This might explain why the amount of S based on modal mineralogy is higher than the S detected from the EDS sum spectra and/or the analytical total S in the case of some samples e.g., 21, 24, 25, 27, and 31. On the other hand, the samples with the total S based on SEM modal mineralogy > 50% lower than the analytical total S (1–2, 4, 6, 13–19, 26, 28, 30, 32, and 34) had an average of analytical total S of 0.51 wt%. This is clearly lower than the average analytical total S of 1.67 wt% of the samples that had the total S based SEM modal mineralogy closer (< 50 wt% lower) to the analytical total S. This indicates that there is an elevated risk (but again, not certainty) that the total S based on SEM modal mineralogy is underestimated if the amount of S-bearing minerals is low. Furthermore, of the 16 samples that had a total S based on SEM modal mineralogy > 50% lower than the analytical total S, 11 (1–2, 4, 13–17, 30, 32, and 34) contained elevated (> 9 wt%) amounts of serpentine, diopside, augite, and/or hornblende, a phenomenon which should be further investigated. In the case of serpentine and hornblende, this might be due to the light element H of the minerals, which cannot be detected by the equipment and may cause uncertainty to the FE-SEM-EDS analysis (Schulz et al., 2020). Another reason could be that the occurrence of very small particle size of sulfides is somehow related to rock types including serpentine, diopside, augite, and/or hornblende. However, this phenomenon should be investigated further.

The determination of AP by mineralogical calculation is in general considered an efficient method in the AMD prediction (Dold, 2017; Karlsson et al., 2018), since the minerals responsible for AP and NP potentials can be identified through mineralogical inspection, and the data can then be utilized in the assessment of the kinetics of the AMD generating processes. Our results show, however, that there is a need to improve the accuracy of the SEM modal mineralogy-based calculations. This could be done by checking the EDS data to verify if the EDS sum spectral S is in line with the modal mineralogy calculation, when calculating the S content based on the SEM modal mineralogy. If the S calculated by the SEM modal mineralogy appears to underestimate the amount of S, normalization of the modal mineralogy to EDS sum spectral S could be made to avoid the underestimation of AP.

Like the modal mineralogy-based S method, the analytical sulfide S determination by lower temperature combustion did not measure all sulfidic S in the samples, which might lead to an underestimation of AP. The assumption in this method is that Fe-sulfides can be vaporized in lower temperatures, in which the other sulfides and sulfate S are not vaporized (Price, 2009; SFS-EN, 2012). This is because the base metal sulfides, especially pentlandite, Cu-sulfides, galena, and sphalerite, have higher thermal stabilities compared with Fe-sulfides (Bucknam, 1999; Lapakko, 2002; Li et al., 2007). This S method is similar to the sulfide sulfur determination method ASTM E 1915, in which the sample is roasted at a temperature of 550 °C or 650 °C (ASTM, 2007). Our results are in line with this assumption, since the analytical sulfidic S/total S ratios were low in the samples with baryte or base metal sulfides chalcopyrite (samples 13, 14, 15, and 18) or pentlandite (sample 27) as the primary sulfide mineral (Table 3, Fig. 2). However, our results further suggest that in contrast with the assumption of the sulfidic S method, Fe-sulfides, neither pyrite nor pyrrhotite, volatilize completely during the low temperature combustion (Fig. 2). As pyrrhotite is the most common sulfide mineral in Finnish mine waste and responsible for the most AP (Karlsson et al., 2018), the use of this method is not recommended as the amount of AP might be underestimated.

Based on the results, the AR extractable S concentrations can be used for a preliminary AMD prediction as an optional method for the analytical total S. The AR extractable S values were in the same order as the analytical total S values (Fig. 1D). The main difference between these methods is that AR does not dissolve barite, which is beneficial for the AMD prediction compared with the use of the analytical total S method. However, especially for samples with > 3 wt% S, the AR extractable S was in our results systematically lower than the analytical total S, which should be considered in the preliminary AMD assessment. Compared with the analytical total S method, lower S concentrations measured with ICP-based methods have also been reported by Ackerman et al. (2012) and Okai et al. (2007). Okai et al. (2007) considered that this might be due to the incomplete decomposition of samples and/or the loss of S in the form of H_2_S during the analysis. As the AR extractable S values were close to the analytical total S values and the AR method is commonly used in exploration (Fletcher, 1981; Koljonen, et al., 1992), additional S analyses with special sample preparation might not be needed aside with the AR method for the preliminary AMD prediction. This could increase the cost-effectiveness of the AMD prediction in an early phase of the mining operations. Furthermore, the AR method is also useful in the mobility assessment of harmful elements (Karlsson, Alakangas, et al., 2021). Another sulfide specific extraction method commonly used in ore exploration is the hydrogen peroxide ammonium citrate extraction (Katsnelson & Osipova, 1960). However, according to Karlsson, Räisänen, et al. (2021), hydrogen peroxide does not decompose Fe-sulfides completely, and some S is lost during the extraction process possibly due to formation of H_2_S, and thereby, the method might underestimate the AP in the preliminary AMD prediction.

In contrast to the AR extractable S, the S values obtained by the XRF method seemed less useful in the AMD prediction than the other methods. This is because the XRF measured S values were on average lower compared with the S values obtained with the other investigated methods, which might result in the underestimation of the AP potential. This was expected, as the XRF method is not highly accurate for S analysis, as some amounts of S is lost by volatilization during the measurement (Norrish & Thompson, 1990; Punkkinen et al., 2021). However, according to Chubarov et al. (2016), the XRF method could be used to estimate the ratio between sulfide and total S in sulfide ores. This technique would involve utilizing the influence of S chemical state on positions and intensities of lines (SKα1,2, SKβ1,3), and satellites (SKβ′, SKα3,4) of the S X-ray emission spectra measured by the wavelength-dispersive XRF spectrometer. The assessment of the usability of this method in the preliminary characterization of waste rock should be further investigated.

Various other total S and sulfidic S analysis methods exist (Ackerman et al., 2012; Lapakko, 2002; Mahanta et al., 2017; Okai et al., 2007; SFS-EN, 2012; Wilson et al., 1963; Yin & Catalan, 2003) that were not investigated in this study. In general, they include the determination of total S with more complicated extractions, or the determination of sulfur species by treating samples to remove a specific sulfur phase and using the total S of the original and treated sample to calculate the S change resulting from the extraction (Lapakko, 2002). These methods are more complicated and require additional work compared with the methods used also in ore exploration, which makes them more expensive and less suitable for the preliminary screening of large number of samples. In addition, extraction methods are rarely 100% mineral-specific (Hall et al., 1996; Heikkinen & Räisänen, 2009; McCarty et al., 1998), which increases the uncertainty of S calculations based on these methods.

An emerging waste rock characterization method and a potential substituent for an early-phase geochemical S-analyses is the automated identification of sulfides and AMD potential assessment from a drill core imagery. For example, Cracknell et al. (2018) utilized successfully red–green–blue images in a combination with hyperspectral data to detect Fe-sulfides. However, they concluded that the geochemical analyses should still be used together with the imagery based automated classification of the AMD potential. Nevertheless, as pointed out by Cracknell et al. (2018), the evolution of the core scanning technology might lead to a substitution of static testing by the new emerging imagery-based methods.

## Conclusions

Pyrrhotite was the most abundant sulfide mineral in the investigated Finnish mine waste, followed by pyrite. Of the other base-metal sulfides, chalcopyrite was the most common mineral, followed by pentlandite and sphalerite. The abundance of sulfide species other than pyrite suggests that the factor to calculate the AP by multiplying the S content should be considered based on the dominating sulfide species, as the amount of H^+^ released in the oxidation processes varies for different sulfides. According to our results, in most of the Finnish mine waste cases, the factor should be lower than the commonly used 31.25. Although S-bearing minerals excluding sulfides and oxidized Fe-sulfides were relatively uncommon or appeared usually in insignificant amounts in the inspected samples, the possible presence of barite should be considered when analyzing S from the mine wastes. When investigating fresh Finnish waste rock samples, the total S values mainly present sulfidic S concentrations, excluding barite-bearing cases. When investigating weathered mine waste material by geochemical analysis methods, the presence of oxidized Fe-sulfides should be considered.

As the mineralogy of the sample affects the calculation factor for AP, a mineralogy-based determination of S species should be preferred. However, the determination of S based on SEM modal mineralogy appears to include some uncertainties. For example, underestimation of S content and AP may occur, if the modal mineralogical analysis has not detected all S-bearing mineral particles, e.g., due to too small grain size or S being bound in trace element contents (below LOD) to certain mineral phases. Furthermore, based on our results, there is an elevated risk of underestimating the SEM mineralogy-based S content if the amount of S is low in the sample. This risk is high especially related to samples containing elevated (> 9 wt%) amounts of serpentine, diopside, augite, and/or hornblende. On the contrary, there is an elevated risk of overestimating the SEM modal mineralogy -based S if the content of S-bearing minerals is high (> 4.13 wt%). The uncertainties related to the SEM modal mineralogy -based S calculation can be reduced by inspecting the EDS spectral data to verify that the S concentration calculated by modal mineralogy is in line with the total S based on the EDS sum spectra, which was noticed to be more in line with the analytical total S values.

Based on our comparison of the different geochemical S analysis methods, the AR extractable S concentrations, which are often available already in the exploration phase, appeared to be usable in the preliminary waste rock AP assessment. The AR extractable S values were comparable with the analytical total S values, especially when the S values were < 3 wt%. Furthermore, unlike the analytical total S analysis, the AR extraction does not include barite, which is beneficial for the AMD prediction. Of the other methods, especially the analytical sulfide S and the XRF method may lead to an underestimation of AP.

## Data Availability

The datasets generated during and/or analyzed during this study are available from the corresponding author on reasonable request.
